# Paradoxical psoriasiform reactions in hidradenitis suppurativa

**DOI:** 10.1097/JW9.0000000000000297

**Published:** 2026-11-03

**Authors:** Bruna G.O. Wafae, Sophio Kirimlishvili, Elene Tsopurashvili, Sydney Look Why, Anusha M. Kumar, Aleena Bacorro, Alexa B. Kimball, Martina L. Porter

**Affiliations:** a Department of Dermatology, Clinical Laboratory for Epidemiology and Applied Research in Skin (CLEARS), Beth Israel Deaconess Medical Center, Boston, Massachusetts; b Harvard Medical School, Boston, Massachusetts

**Keywords:** Biological therapy, epidemiology, hidradenitis suppurativa, immunology, psoriasis

**What is known about this subject in regard to women and their families?**

- Biologic medications are central to the management of hidradenitis suppurativa (HS), but they can sometimes trigger side effects known as paradoxical psoriasiform reactions (PPR).
- Because HS itself is more common in women, female patients are the demographic most frequently encountering these biologic-induced reactions.

**What is new from this article as messages for women and their families?**

- Women and families should be aware that approximately 10% of patients receiving biologics for HS develop these psoriasiform reactions, a rate that exceeds what is typically seen in other immune-mediated diseases.
- While historically rare, IL-17 inhibitors actually carry the highest risk for triggering these reactions in HS patients compared to other biologic classes.
- Although about one-third of patients may ultimately need to stop their biologic medication due to the reaction, the majority of these rashes can be successfully managed without halting HS therapy.

Dear Editor,

Biologic therapy for hidradenitis suppurativa (HS) management can trigger paradoxical reactions. Paradoxical psoriasiform reactions (PPR) are well documented with tumor necrosis factor alpha (TNF-α) inhibition and have a distinct pathophysiology, mediated by interferon-α.^1^ Interleukin-17 (IL-17) inhibitors are linked to eczematous and other cutaneous reactions.^2^ Nevertheless, the occurrence of PPRs in HS has not been well characterized.

This single-center retrospective study quantified PPR incidence, features, and management in biologic-treated adults (≥18 years) with HS (January 2023–May 2024). Dermatologist-diagnosed PPR was defined by new-onset or worsening psoriasiform lesions temporally associated with biologic exposure and the exclusion of alternative etiologies. Overall cumulative incidence was calculated at the patient level; drug-specific incidences at the exposure level. Exposure time spanned biologic initiation to reaction onset or last follow-up.

Of 222 biologic-treated patients with HS (288 exposures), 22 (23 reactions) developed PPRs, yielding a 9.9% cumulative incidence (22/222; 95% confidence interval = 6–13.8%) over ≤16 months. One reacted to adalimumab and bimekizumab. Cases were mostly female (72.7%; 16/22), white (45.5%; 10/22), and Hurley stage III (54.5%; 12/22). Compared to non-PPR patients, cases were older (mean [SD] age = 45.4 [14.3] vs 39.7 [12.9]; *P* = .055), had more prior psoriasis/psoriatic arthritis (31.8% vs 11%; *P* = .053), and were more frequently exposed to IL-17 inhibitors (45.5% vs 31.5%; *P* = .055); though non-significant (Table 1).

**Table 1.** Baseline characteristics by PPR group

	Total patients (222)	Patients without paradoxical psoriasis (200)	Patients with paradoxical psoriasis (22)	*P* value
Age, mean (SD)	40.33 (13.1)	39.77 (12.9)	45.41 (14.3)	.055
Gender, *n* (%)				
Female	156 (70.3)	140 (70.0)	16 (72.7)	.921
Male	65 (29.3)	59 (29.5)	6 (27.3)	
Race, *n* (%)				
Asian	10 (4.5)	10 (5.0)	0 (0.0)	.778
Black	55 (24.8)	48 (24.0)	7 (31.8)	
White	111 (50.0)	101 (50.5)	10 (45.5)	
Other	20 (9.0)	18 (9.0)	2 (9.1)	
Unknown	26 (11.7)	23 (11.5)	3 (13.6)	
Hurley stage, *n* (%)				
I	20 (9.9)	19 (10.5)	1 (4.5)	.176
II	108 (53.2)	99 (54.7)	9 (40.9)	
III	75 (36.9)	63 (34.8)	12 (54.5)	
Prior psoriasis, *n* (%)				
Yes	29 (13.1)	22 (11.0)	7 (31.8)	.053
No	191 (86)	176 (88.0)	15 (68.2)	
Biologic class				
TNF inhibitors	189 (85.1)	176 (88.0)	13 (59.1)	.055
IL-17 inhibitors	73 (32.9)	63 (31.5)	10 (45.5)	
IL-23 inhibitors	26 (11.7)	26 (13.0)	0 (0)	

Comparisons employed Mann–Whitney U or χ^2^/Fisher exact tests.

IL, interleukin; TNF, tumor necrosis factor.

IL-17 inhibitors were associated with the highest risk of PPR at 13.7% (10/73) (bimekizumab 4/15 [26.7%], ixekizumab 1/4 [25%], and secukinumab 5/53 [9.4%]), followed by TNF-α-inhibitors at 6.9% (13/189) (infliximab 4/35 [11.4%] and adalimumab 9/149 [6%]); no PPRs occurred with IL-23/IL-12/23 inhibition. TNF-induced PPRs developed after a median of 15.1 months (interquartile range [IQR] = 5.8–50.1); 61.5% after dose escalation. IL-17-induced PPRs appeared sooner (3.3 months [IQR = 1.5–7.70]). Morphology was similar across classes: inverse distribution predominated (56.5%); palmoplantar and scalp involvement each occurred in 17.4% (Fig. 1). Management was mainly topical (60.9% topical steroids/calcineurin inhibitors). Systemic therapy was used in 21.5% (JAK inhibitor 17.4%; apremilast/methotrexate 2%) and 34.8% necessitated discontinuation of the index biologic.

**Fig. 1.**
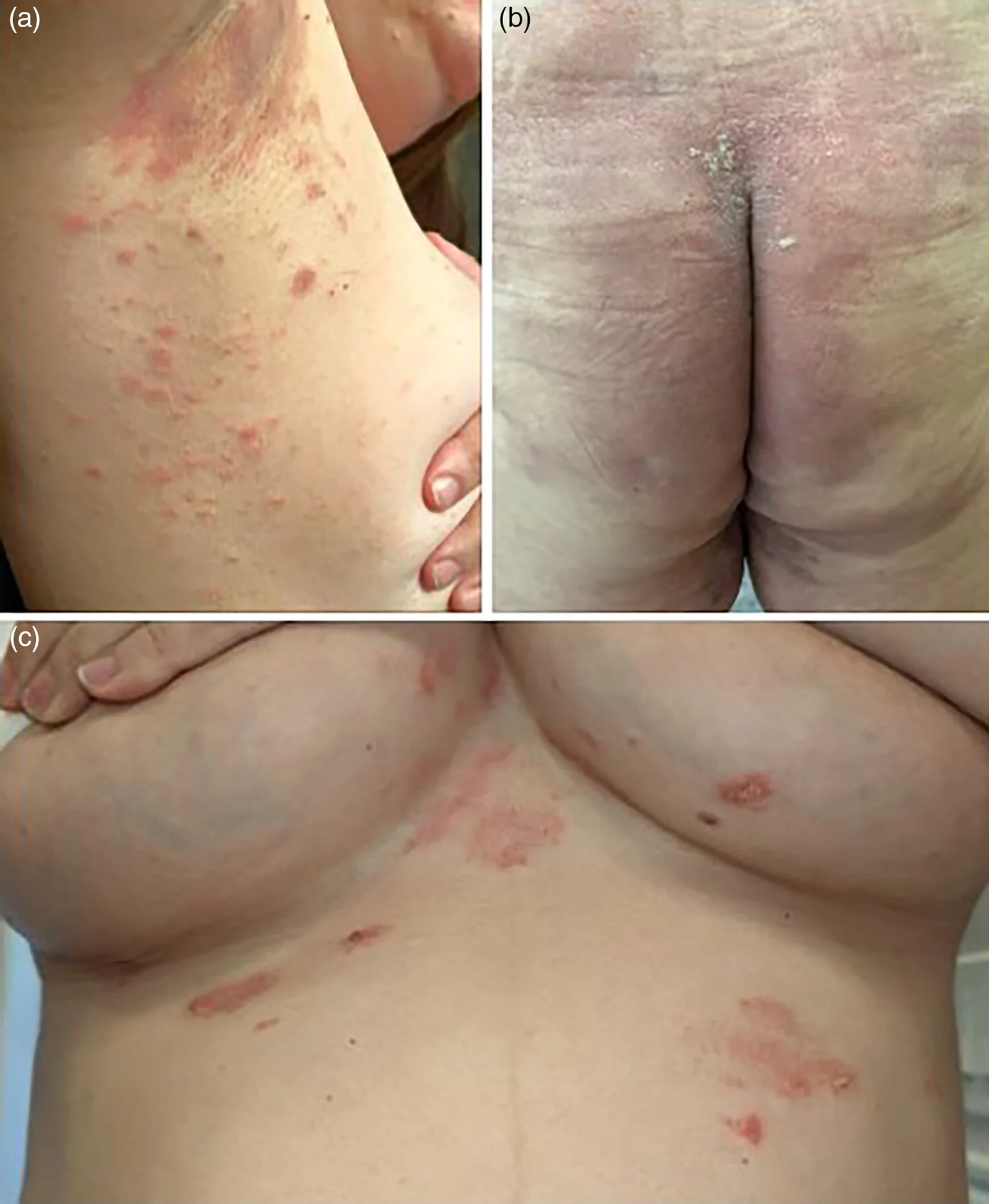
Clinical presentation of paradoxical psoriasiform reactions in our cohort. (A) Well-demarcated erythematous plaque in the right axilla associated with peripheral 1–3 mm satellite papules. (B) Symmetric erythematous plaques on bilateral buttock cheeks with fine scale accentuated at the superior gluteal cleft. (C) Multiple nummular 10–30 mm erythematous, scaly plaques on the inframammary folds and intermammary.

In this cohort, 9.9% of biologic-treated HS patients developed PPR, which exceeds proportions reported for other immune-mediated diseases, despite different study designs.^3^ While HS carries a known baseline association with psoriasis, background prevalence is typically lower (2–4%) and rarely presents as inverse disease. IL-17 inhibitors, rarely associated with PPR historically, showed the highest cumulative incidence. Long-term safety data in HS are still emerging, and the exact cause of this higher incidence remains unknown.^3^ Clinically, inverse distribution predominated, unlike the palmoplantar pustular pattern previously reported in 41–60% of TNF-α–related PPRs,^3–5^ suggesting PPR phenotype may be modulated by the underlying cytokine milieu and HS anatomic predilections.

Lesions typically manifested as erythematous scaly plaques in intertriginous sites with occasional maceration and satellite papules, mimicking candidiasis or irritant dermatitis. Prior PsO/PsA in one-third supports a potential risk factor.^3^ Most eruptions responded to topical therapies. Conventional systemic therapies such as methotrexate and cyclosporine have limited efficacy and side effects.^4^ Nevertheless, 34.8% required discontinuation of the culprit biologic. Decisions should weigh HS control against quality-of-life impact, which can be underestimated by the low body-surface-area in special sites. When switching is necessary, cycling to another TNF agent carries a high recurrence risk^4^; selecting an alternative mechanism (e.g., IL-17-inhibitors, upadacitinib, currently in phase III HS trials, or ustekinumab, efficacious for TNF-induced psoriasis in IBD) may be preferable.^4^

Limitations include the retrospective design, limited statistical power, lack of a non-biologic control, and modest sample size. Future prospective studies utilizing standardized criteria and histopathological confirmation are needed to distinguish PPR from clinical mimics and prevent overdiagnosis. PPRs are not uncommon in biologic-treated HS, and often show inverse morphology. Awareness supports counseling, monitoring, and timely treatment adjustment.

## Conflicts of interest

The authors made the following disclosures: Dr B.G.O.W. is an investigator for AbbVie, Eli Lilly, Incyte, Moonlake, Avalo, Prometheus, Sanofi, Insmed, UCB, Novartis, Pfizer, and Bristol Meyers Squib. Dr A.M.K. reports receiving personal fees (advisory board) from Boehringer Ingelheim. Dr M.L.P. received consulting fees from AbbVie, Alumis, Arcutis, Avalo, Eli Lilly, FIDE, Incyte, Janssen, Merck, MoonLake Immunotherapeutics AG, Navigator Biosciences, Novartis, Pfizer, Sanofi, Sonoma Biotherapeutic, Trifecta Clinical/WCG, UCB, and ZuraBio; serves as chair for the American Academy of Dermatology (AAD) patient safety and quality committee and the Symposium on Hidradenitis Suppurativa Advances planning committee; is a member of the AAD Science and Research Council; and has received royalties for the BIDMC training module. Dr M.L.P.’s institution has received grants from AbbVie, AnaptysBio, Avalo, Bayer, BMS, Eli Lilly, Incyte, Janssen Pharmaceuticals, MoonLake, Immunotherapeutics AG, Novartis, Oasis Pharmaceuticals, Pfizer, Prometheus Laboratories, Regeneron, Sonoma Biotherapeutics, Sanofi, and UCB. Dr A.B.K. institution has received grants from Anapyts Bio, Bristol Myers Squibb, Eli Lilly, Incyte, Janssen, Moonlake, Novartis, Pfizer, Prometheus, Sanofi, Sonoma Bio, UCB, Regeneron, Avalo, Acelyrn; consulting fees from Abbvie, Eli Lilly, Janssen, Moonlake, Novartis, UCB, Boehringer Ingelheim, Takeda, Avalo, Cellarity, Citryll, Evoimmune, Merck, Pfizer, Sanofi, Sonoma Bio, Target RWE, Nurix, Union Therapeutics, Ventyx, Zura bio, Board of Directors Almirall. No other disclosures were reported.

## Funding

None.

## Study approval

This study was reviewed and approved by the BIDMC IRB.

## Author contributions

BGOW: Participated in conceptualization, data curation, formal analysis, investigation, methodology, software, visualization, writing – original draft, and writing – review & editing. SK, ET, SLW, and AB: Participated in data curation, formal analysis, investigation, visualization, writing – original draft, and writing – review & editing. AMK: Participated in investigation, methodology, visualization, writing – original draft, and writing – review & editing. ABK and MLP: Participated in conceptualization, formal analysis, investigation, methodology, project administration, resources, software, supervision, visualization, writing – original draft, and writing – review & editing.

## Data availability statement

The data that support the findings of this study are available on request from the corresponding author. The data are not publicly available due to privacy or ethical restrictions.

## Ethics approval

Reviewed and approved by BIDMC IRB. The patients in this manuscript have given written informed consent to publication of their case details.
